# The transmembrane replacement H7N9-VLP vaccine displays high levels of protection in mice

**DOI:** 10.3389/fmicb.2022.1003714

**Published:** 2022-10-06

**Authors:** Jianru Qin, Bing Hu, Qiqi Song, Ruijuan Wang, Xiangfei Zhang, Yaqi Yu, Jian-Hua Wang

**Affiliations:** ^1^College of Life Sciences, Henan Normal University, Xinxiang, China; ^2^Guangzhou Institutes of Biomedicine and Health, Chinese Academy of Sciences, Guangzhou, China

**Keywords:** H7N9 influenza virus, passive immunity, vaccine, virus-like particles, BALB/c

## Abstract

The incidence of infections caused by the H7N9 subtype of the influenza virus has expanded rapidly in China in recent decades, generating massive economic loss and posing a significant threat to public health. In the absence of specialized antiviral treatments or long-term effective preventative vaccinations, it is critical to constantly enhance vaccines and create effective antiviral drugs to prevent the recurrence of pandemics. In the present study, a transmembrane-substituted (TM) virus-like particle (VLP)-based vaccine was created by replacing the transmembrane region of hemagglutinin (HA) protein with the transmembrane region of the H3 HA protein and then used to immunize BALB/c mice. Sera and T cells were collected from the immunized mice to evaluate the passive immune effects. Our results showed that naïve mice achieved 80–100% protection against homologous and heterologous H7N9 influenza strains after receiving passive serum immunization; the protective effect of the TM VLPs was more evident than that of the wild-type HA VLPs. In contrast, mice immunized with passive T cells achieved only 20 to 80% protection against homologous or heterologous strains. Our findings significantly contribute to understanding the control of the H7N9 virus and the development of a vaccine.

## Introduction

China has experienced five outbreaks since the first human infection with the H7N9 avian influenza virus in February 2013 (Shen and Lu, 2017). Patients infected with H7N9 develop acute respiratory distress syndrome. The case fatality rate is approximately 38%, although some patients can have mild symptoms or be asymptomatic (Chen et al., 2013, 2014; Yi et al., 2015). H7N9 is an extraordinary and ongoing issue that threatens public health and the poultry industry, necessitating immediate and effective prophylactic and therapeutic measures (Gao et al., 2013; Shen and Lu, 2017).

Vaccination is the most direct and successful method for preventing H7N9 infections, and several types of vaccines have been produced, including inactivated, live-attenuated, DNA, subunit, and recombinant vector vaccines (Stephenson et al., 2004; Francis, 2018). Trivalent/quadrivalent inactivated or attenuated vaccines are currently used for influenza vaccinations (Qin et al., 2018). The manufacturing process for those vaccines possesses several limitations, particularly the long manufacturing time that makes meeting vaccine preparation requirements during rapid outbreaks difficult (Gerdil, 2003; Duque et al., 2011). The selection of anti-influenza drugs is limited for pharmacotherapeutics. The approved M2 ion channel inhibitors are only effective against influenza A, which is already widely resistant (Houser and Subbarao, 2015). In addition, neuraminidase inhibitors have obvious side effects and are not efficacious in severe cases. H7N9 is resistant to amantadine and oseltamivir causes severe infection in humans by rapidly inducing progressive acute community-acquired pneumonia, multiple organ failure, and cytokine dysregulation (Wang et al., 2020).

In the absence of specific antiviral drugs or sustained and effective preventive vaccines, passive immunotherapy with recombinant antibodies from single-donor plasma or combined immunoglobulin products is also an effective approach, with a good safety and efficacy record against viruses including respiratory tract viruses (Casadevall and Pirofski, 2020; Boonyaratanakornkit et al., 2021). Methods for the treatment and prevention of infectious illnesses based on immune serum-mediated humoral immunity and immune T cell-mediated immunity have been shown to be beneficial. The strategy of passive immunization by injecting immune serum from immunized (or previously infected) individuals has made considerable clinical progress in recent years and has been used to prevent infections by influenza viruses (Kalenik et al., 2014), human papillomavirus (HPV; Kwak et al., 2011), human immunodeficiency virus (HIV; Hsu and O'Connell, 2017), and rabies virus (Both et al., 2012), among others. The main advantages of passive immunization include its wide coverage, strong tolerance, good efficacy, rapid protection, and high economic benefit against multiple virus strains or serotypes. Current research is aimed at developing candidate vaccines that can confer effective and protective passive immunity against influenza.

Previous studies have indicated that replacement of the transmembrane region of the hemagglutinin (HA) protein from the H1, H5, H7, or H9 influenza virus subtypes with the H3 subtype can increase the stability of the protein or virus and improve immunogenicity (Wang et al., 2017; Zhang et al., 2017). These results demonstrated that replacement of the TMs of non-H3 HAs with H3-WT TM could enhance their hetero-protection (Liu et al., 2014). In the present study, the production of immune serum and T cells by virus-like particles (VLPs) containing the transmembrane replacement region were used for the passive immune challenge. The VLPs composed of wildtype (WT) HA or transmembrane-substituted (TM) HA and matrix protein 1 (M1) showed excellent protective effects in serum passive immunity challenges. This study presents a new concept for the development of universal protection from influenza through humoral and cellular passive immunity acquired *via* influenza virion vaccinations; it is expected to form the basis of a reasonable and effective candidate vaccine for H7N9 influenza to relieve public health pressures. The prospects of cellular and humoral passive immunization in vaccine development are emphasized.

## Materials and methods

### Viruses and cells

Two H7N9 virus strains were used in this study to evaluate serum and T-cell passive immunity: A/Chicken/Guangdong/53/2014(H7N9; H7N9–53) and A/Chicken/Guangdong/MCX/2014(H7N9; H7N9-MCX). These isolates were derived from chickens in Guangdong, China, belonging to different branches of the same Yangtze River Delta (YRD) lineage and stored in our laboratory. The amino acid sequences similarity between the two strains was 98.21% with the exception of the mutation at residue 186, also differed at 9 amino acid sites, which may have an impact on the receptor binding capacity of the virus (The GeneBank accession numbers corresponding to H7N9–53 virus is KY221841; H7N9-MCX is KY221844).

Madin-Darby canine kidney (MDCK) cells were cultured in Dulbecco’s Modified Eagle’s Medium (DMEM, Gibco, NY) containing 10% fetal bovine serum (FBS, Gibco, NY) and were used in the assessment of the viral load of the H7N9 influenza virus.

### Production of H7N9 VLPs-WT/TM and mouse vaccinations

In this study, we explored and compared the immunological characteristics of two H7N9 VLPs constructed from M1 and WT HA or TM HA replaced with H3N2. These two-component VLPs derived from the H7N9–53 virus, H7N9 VLPs-WT and H7N9 VLPs-TM, were purified by ultracentrifugation through sucrose density gradients and expressed using an insect baculovirus expression system, as previously described (Qin et al., 2018).

**Figure 1:**
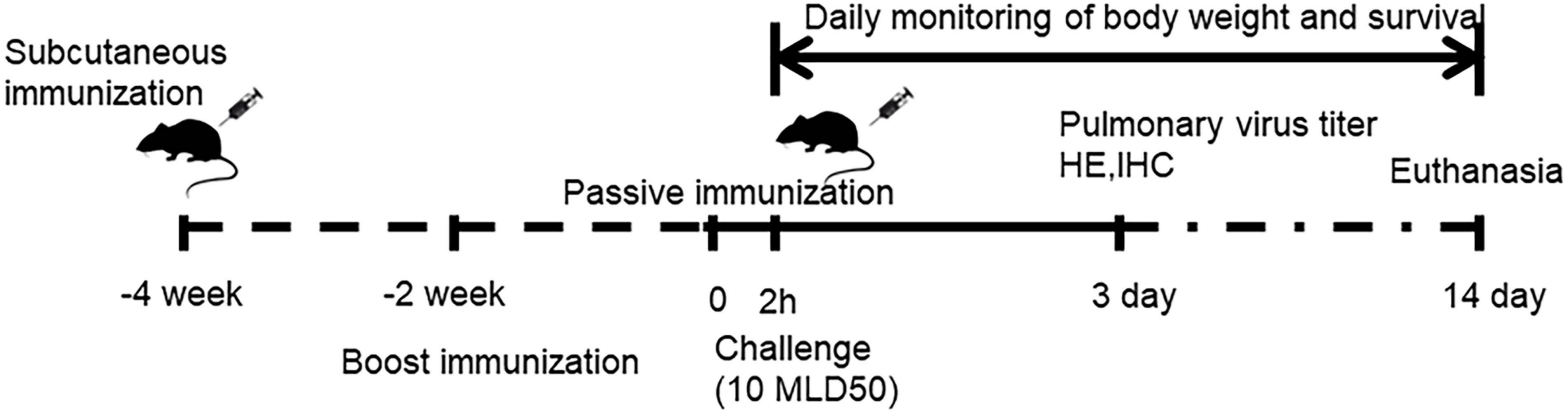
Schedule of immunization, sampling and passive immunization effect evaluation in mice.

Thirty-six female BALB/c mice, aged 6–7 weeks, were randomly allocated into three groups (n = 12 per group) that received H7N9 VLPs-WT, H7N9 VLPs-TM, or phosphate-buffered saline (PBS). Briefly, the mice were prime-boosted with 50 μl VLPs containing 1 μg of HA subcutaneously at two-week intervals. Two weeks after the boost, serum or T cells were collected for evaluation of passive transfer protection (Figure 1).

### Passive transfer and challenge experiment

To assess the functional effect of humoral and cellular immunity in protecting mice from homologous and heterologous H7N9 viruses, serum and T cell passive transfer experiments were performed in 6-week-old female mice. Briefly, the same volume of collected sera from the immunized mice in each treatment group was pooled, followed by heat inactivation at 56°C for 30 min and storage at −80°C until transfer. T cells were purified from spleens isolated from the immunized mice using the Pan T Cell Isolation Kit II (MACS Miltenyi Biotec, Bergisch-Gladbach, Germany) and tested by flow cytometry. The biotin-labeled antibodies mainly included CD11b, CD11c, CD19, CD45R (B220), CD49b (DX5), CD105, anti-MHC class II, and TER-119. The T cell concentration was adjusted to 2 × 10^7^ cells/mL. Naïve mice (a total of 48, *n* = 8 per group) were injected intraperitoneally with the thawed serum (200 μl) or intravenously with the collected T cells (100 μl, 2 × 10^7^ cells/mL). Two hours after the passive transfer, the immunized mice were challenged intranasally with 10× the median lethal dose (MLD_50_) of H7N9–53 or H7N9-MCX virus. Three mice per group were euthanized 3 days after the challenge to measure lung viral load, and the remaining five mice per group were observed for 14 days for weight loss and survival. An infected mouse that lost >25% of its initial body weight was humanely euthanized and considered dead.

### Pulmonary viral load determination

Three days after the challenge, the viral load in the mice lungs was measured by a Madin–Darby Canine Kidney (MDCK) cell plaque test (Zhou et al., 2014). The lowest detection drop in the virus was detected at a titer of 10^1^ PFU/lung; lung samples lower than this titer were assigned a value of 10^0.5^ PFU/lung, which is the effective detection limit.

### Examination of histopathology and immunohistochemistry (IHC)

To evaluate the protection of mice receiving different passive immunizations against the intranasal challenge of homologous or heterologous H7N9 virus strains, 3 days after the challenge, the lung tissues of the mice (*n* = 3 per group) were collected to evaluate lung lesions by histopathology and IHC. Before microscopic observation, the tissues were fixed with formalin, embedded in paraffin, and stained with hematoxylin and eosin (H&E). Examination of influenza viral antigen in the lungs was performed by IHC analysis using an anti-influenza nucleoprotein (NP) antibody (Huo et al., 2017).

### Statistical analysis

Statistical analysis was performed using GraphPad Prism 8. Analysis of Variance (ANOVA) followed by Dunnett’s multiple comparison tests were used for statistical comparisons. Results with *p* values <0.05 were considered statistically significant.

## Results

### Passive transfer of sera and adoptive transfer of T cells can improve overall survival and reduce weight loss

To verify the protection of H7N9 VLPs-WT/TM afforded by humoral immunity and cellular immunity, serum, and immunocytes from the immunized mice were collected and transferred into naïve mice 2 weeks after the boost vaccination of the donor mice. The recipient mice were subsequently challenged with 10 MLD_50_ of H7N9–53 or H7N9-MCX.

**Figure 2:**
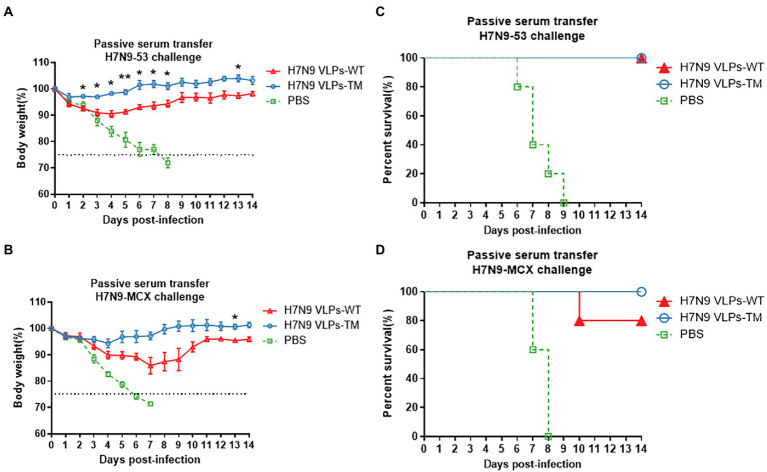
Protection of mice passively immunized with sera and then challenged with homologous and heterologous viruses. **(A)** Weight loss of challenged with H7N9-53. **(B)** Weight loss of challenged with H7N9-MCX. **(C)** Survival rate of challenged with H7N9-53. **(D)** Survival rate of challenged with H7N9-MCX. Statistical difference between two groups was indicated by *p < 0.05, **p < 0.01, ***p < 0.001.

Compared to H7N9 VLPs-WT, sera passive immunization with H7N9 VLPs-TM showed less body weight loss, regardless of the challenge with the homologous or heterologous H7N9 viruses (Figures 2A,B). There was complete protection with passive transfer of H7N9 VLPs-WT/TM sera after the challenge with the homologous H7N9 virus H7N9–53. The body weight of the PBS group decreased rapidly to less than 70%. The survival rate observed after treatment with the H7N9 VLPs-TM sera was 100% after challenge with the heterologous H7N9 virus H7N9-MCX, while that observed after treatment with the H7N9 VLPs-WT sera was 80% (Figures 2C,D).

**Figure 3:**
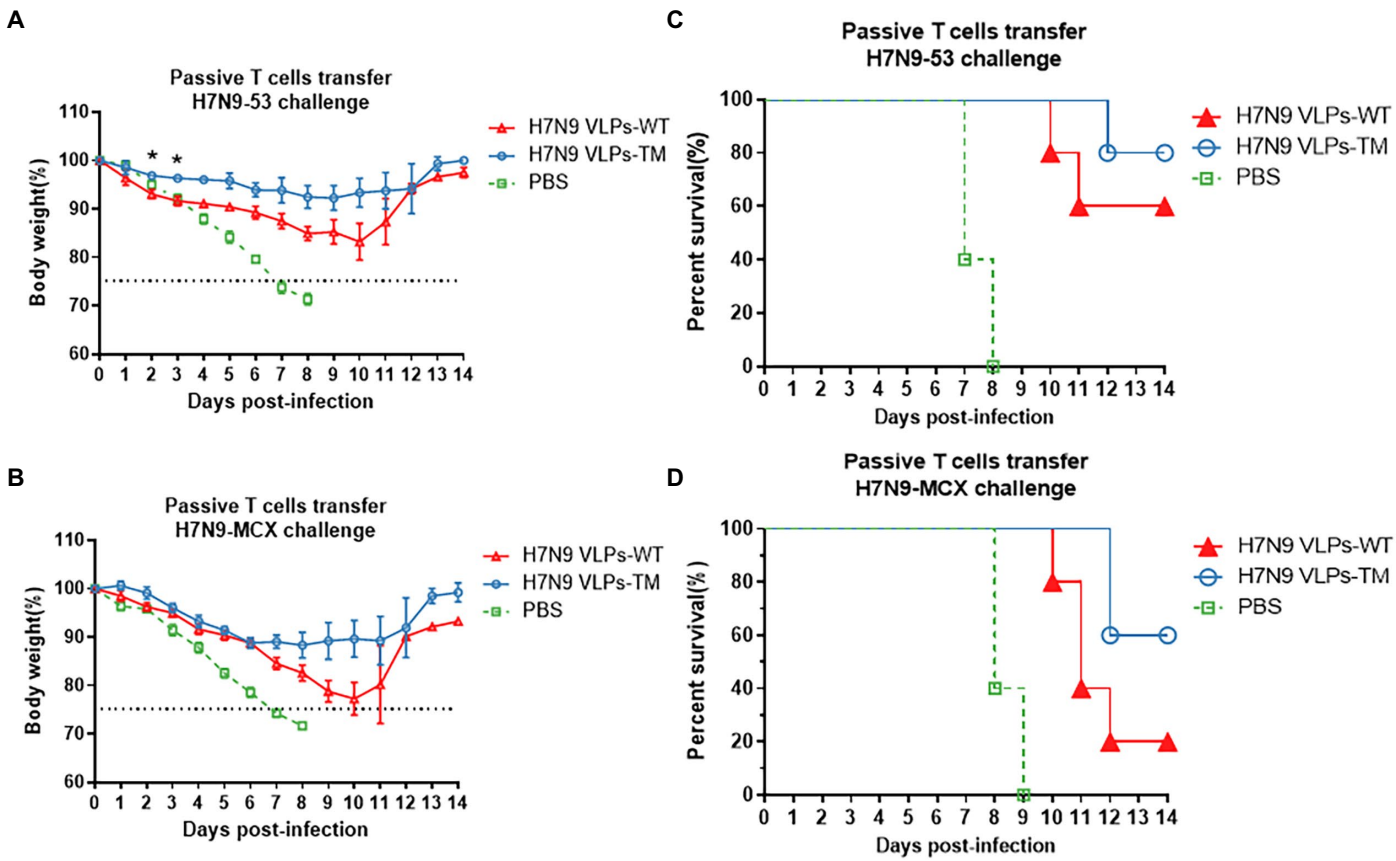
Protection of mice passively immunized with T cells and then challenged with homologous and heterologous viruses. **(A)** Weight loss of challenged with H7N9-53. **(B)** Weight loss of challenged with H7N9-MCX. **(C)** Survival rate of challenged with H7N9-53. **(D)** survival rate of challenged with H7N9-MCX. Statistical difference between two groups was indicated by *p < 0.05, **p < 0.01, ***p < 0.001.

Evident body weight losses were observed in recipients of T cells after the challenge with homologous and heterologous H7N9 viruses (Figures 3A,B). Overall, the reductions in body weight after the H7N9-MCX challenge were higher than that after the H7N9–53 challenge. Moreover, the transfer of T cells from H7N9 VLPs-TM immunized donors resulted in a lower body weight loss in recipients than the transfer of T cells from H7N9 VLPs-WT donors, regardless of the H7N9 viral challenge in the recipients. Transfer of T cells from H7N9 VLPs-WT immunized mice resulted in recipient survival rates of 60 and 20% after the challenge with H7N9–53 and N7N9-MCX, respectively, which were much lower than the survival rates of 80 and 60%, respectively after T cell transfer from H7N9 VLPs-TM immunized mice (Figures 3C,D). The body weight losses in recipient mice after the transfer of T cells from PBS-injected donor mice were the highest of the three groups and the survival rate was the lowest; the same trends were observed with the serum transfer experiment. After passive transfer of sera from PBS-injected donor mice, all recipient mice succumbed to subsequent viral challenge 7–8 days later.

These results indicate that the serum antibodies induced by H7N9 VLPs played an important role in the immunity protection against homologous and heterologous viruses.

### H7N9 VLPs-TM can reduce viral load in the lungs *via* the acquisition of passive immunity

**Figure 4:**
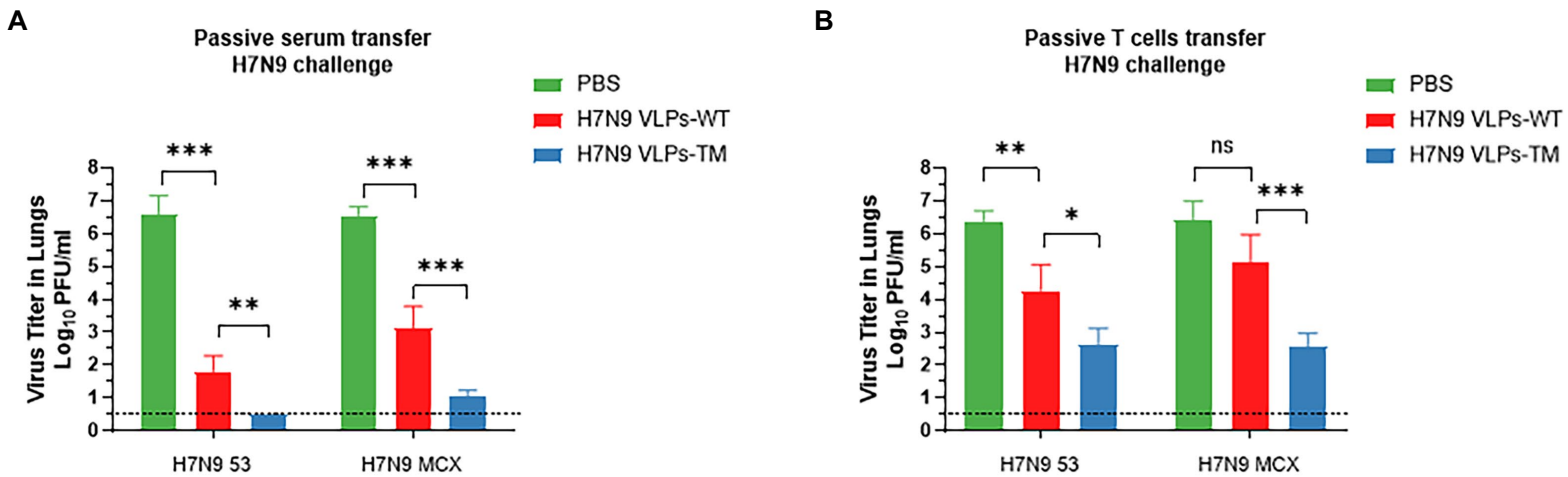
Viral load in lung of mice 3 d after challenge, the titer of influenza virus in the lung tissue was assessed by MDCK cell plaque test and expressed as PFU/ml. The data shown represent the mean + standard deviation (error lines) of three mice per group. **(A)** Passive serum transfer H7N9 challenge. **(B)** Passive T cell transfer H7N9 challenge. The difference of virus titer between the two groups was analyzed by two-way ANOVA, *p < 0.05, **p < 0.01, ***p < 0.001.

After 3 days of challenge, we further evaluated the effects of serum and T cell passive immunization on mice lung viral loads (Figure 4). Serum passive immunization significantly reduced the titer of H7N9–53 or H7N9-MCX virus (Figure 4A). In the H7N9 VLPs-TM immunization group, the infectious virus was below the effective detection limit in the lung tissue after H7N9–53 challenge, which was defined as 10^0.5^ PFU/lung. T cell passive immunization also inhibited the replication of the two viruses; however, there was no significant difference between the H7N9 VLPs-WT immunization group and the PBS group after the H7N9-MCX challenge (Figure 4B). These results indicate that passive immunization with serum or T cells from mice treated with H7N9 VLPs-WT or H7N9 VLPs-TM could reduce the viral load of H7N9–53 or H7N9-MCX in recipient mice. Overall, similarly to previous weight and survival results, serum immunization exhibited the best effect, and the effect of H7N9 VLPs-TM was better than the H7N9 VLPs-WT.

### Passive immunization can prevent lung damage caused by the H7N9 virus

**Figure 5:**
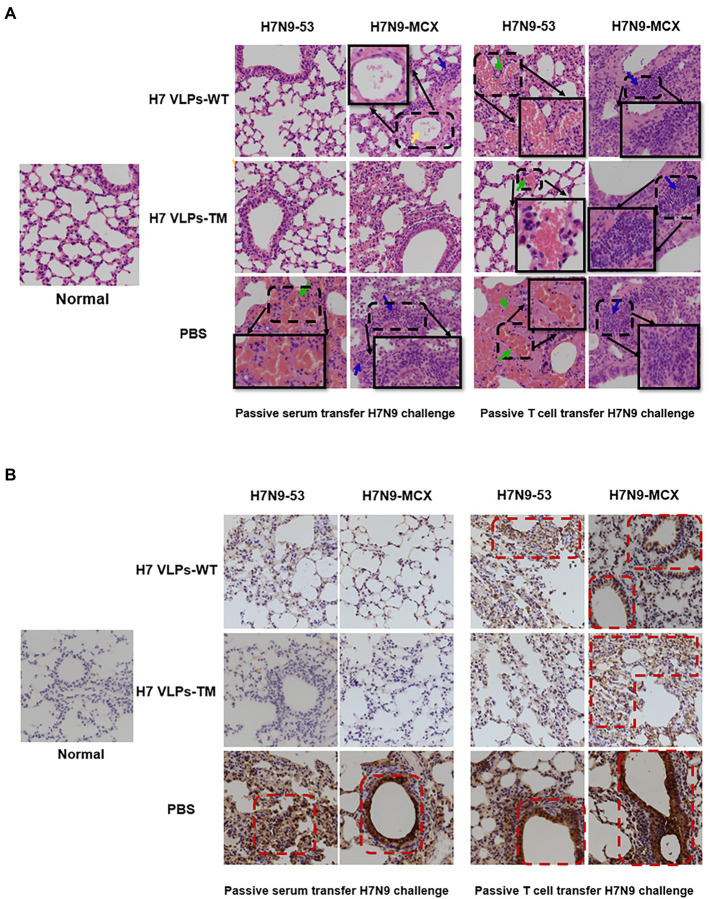
Pathological sections and immunohistochemistry of the lungs of passively immunized mice challenged with poison (400×). **(A)** Histopathology examination (the green arrow represents congestion and bleeding, blue arrows represent lymphocyte infiltration, and yellow arrows represent exfoliated cells in the alveolar cavity). **(B)** Immunohistochemistry showing expression of influenza NP in lung sections. NP, nucleoprotein.

The H&E results showed that passive immunization with serum or T cells from the PBS group led to serious lesions 3 days after viral challenge, with obvious diffuse alveolar injury, narrowing of alveolar cavities, and widening of the alveolar septum. There was evident inflammatory cell infiltration (Figure 5A, blue arrow), congestion, and bleeding (Figure 5A, green arrow). Both the H7N9 VLPs-WT and H7N9 VLPs-TM showed good protective effects against the challenge with the two viruses. In addition, it is worth mentioning that when H7N9-MCX was used, slight lymphocyte infiltration and exfoliated cells were observed in the alveolar cavities in the H7N9 VLPs-WT group (Figure 5A, yellow arrow). In general, serum passive immunization was better than T cell passive immunization, and the protective effect of H7N9 VLPs-TM was better than that of H7N9 VLPs-WT. The protective effect of the two H7N9 VLPs was more obvious against homologous virus H7N9–53 than against heterologous virus H7N9-MCX.

Using immunohistochemistry, we observed diffuse and intense expression of NP in the pulmonary alveoli of the PBS group (Figure 5B). This was consistent with the results of H&E staining. In addition, immunostaining of normal tissue sections was used as a control to exclude the possibility of non-specific staining.

## Discussion

The current influenza vaccine strategies mainly focus on producing a strong antibody response. To confirm this view, in the present study, we evaluated the protective effects of the immunized mice serum and T cells on naïve mice and challenged them with homologous or heterologous strains. The results highlighted the irreplaceable role of the humoral immune response against influenza virus infection. H7N9 VLPs-TM serum showed 100% protection against fatal challenges with homologous or heterologous H7N9 viruses (100% protection against H7N9–53 virus). However, mice passively immunized with the serum against H7N9 VLPs-WT could be completely protected from homologous challenge, and the protection against heterologous challenge reached 80%. In addition, we conducted experiments using T cell passive immunity, and the results demonstrated that the passive protection with H7N9 VLPs-TM T cells against H7N9-MCX and H7N9–53 was 60 and 80%, respectively, while the protection with H7N9 VLPs-WT T cells was 20 and 60%, respectively. These results indicate that humoral immunity plays an important role in protection and that our H7N9 VLPs-TM produced a good protective effect.

In conclusion, we evaluated the protection against homologous or heterologous viruses afforded by passive immunity from sera and T cells of donor mice immunized with VLPs composed of H7N9 HA-WT and M1 or H7N9 HA-TM and M1. Both serum and humoral immunity protected mice against weight loss, mortality, and reduced pulmonary virus titers; however, in the same passive immunity type, H7N9 VLPs-TM was better able to resist challenges with homologous or heterologous viruses. These results highlight the protective role of antibody responses induced by candidate vaccines in H7N9 VLP-induced passive serum immunity. Due to the limited selection of antiviral drugs, passive transferable immune sera have been used as a form of immunotherapy in clinical practice with great success for other viral infections, such as severe acute respiratory syndrome coronavirus 2 (SARS-CoV-2), which began in late 2019 (Pavia and Wormser, 2021), and Ebola (Edwards et al., 2016). At present, human convalescent plasma containing SARS-CoV-2 antibodies is being used to treat coronavirus disease 2019 (COVID-19) patients, and clinical efficacy trials are underway (Cross et al., 2021). Passive immunization is an effective and safe method to combat specific infectious diseases (Raab, 2011). Data from a prior study indicates that passive immunization *via* serum transfer may have clinically relevant effects on reducing mortality and viral load in patients infected with the Spanish H1N1 virus (Mair-Jenkins et al., 2015). A 2009 study revealed that serum passive immunotherapy may play a role in the treatment of patients with severe H1N1 infection, effectively reducing viral load, inhibiting cytokine responses, and reducing mortality (Hung et al., 2011). Based on two H7N9 VLPs composed of HA-WT/TM and M1, the present study presents a new concept for the development of a universal influenza vaccine and contributes to the transformation of vaccine development for clinical development.

Although we have an improved understanding of the passive immunity from influenza VLP vaccines, the host immune response also involves multiple complex processes that persist for long periods. In the present study, the mice were challenged only 2 h after receiving passive immunity, and longer times were not examined. However, the protection of mice against homologous and heterologous strains 2 h after receiving passive immunization was evident, which also reflected the outstanding effect of passive immunization derived from H7N9 VLPs composed of HA-WT/TM and M1. The main purpose of this study was to evaluate the efficacies of the VLPs-TM and VLPs-WT vaccines through the investigation of the protective effects of the passive immunization of mice with these vaccines. The challenge test was performed before specific antibodies were produced, and another set of passive immunization tests could be considered in the later stage to explore the criteria for the production of specific antibodies. In addition, the biggest limitation of this experiment was that the results were not compared to that of those using whole inactivated virus (WIV). However, Choi et al. studied the immunogenicity and protective effect of HA-VLP and WIV antigen in chickens (Choi et al., 2013). The serum antibody level induced by VLP was similar to that induced by WIV antigen; however, the average hemagglutination inhibition (HI) antibody titer increased markedly after intranasal challenge with H5N1 HPAI virus. The serum antibody level of the HA-VLP group was significantly higher than the WIV group in this situation. This indicates that HA-VLP may have a better protective effect than WIV. It is also worth noting that passive immunization with serum during a pandemic does have the expected protective effect, but considering the risk of serum sickness, the use of whole blood for passive immunological assessment is indeed lacking. One inherent limitation of this study was that the mouse model was used to extrapolate human immune reaction response. Recent studies used ferrets as an alternative considering its high susceptibility to influenza virus, similar lung physiology and patterns of binding to sialic acid with human (Belser et al., 2011; Oh and Hurt, 2016). Future studies of this H7N9 vaccine candidate could consider validating its efficacy in a ferret challenge model.

Overall, our study demonstrates that two VLPs of H7N9 composed of HA-WT/TM and M1 induced the production of immune sera and T cells and that the resulting passive immunity acquired by the mice showed good protection against homologous and heterologous viruses. These findings provide a new strategy for the rapid preparation of effective vaccines against influenza pandemics.

## Data availability statement

The original contributions presented in the study are included in the article/supplementary material, further inquiries can be directed to the corresponding author.

## Ethics statement

All experiments were performed in strict accordance with the “Guidelines for The Care and Use of Experimental Animals of the People’s Republic of China.” Approved by the Animal Care and Use Committee of the Henan Normal University (Permit Number: HNSD2020-0207) and strictly abided by research and experimental ethics and relevant regulations.

## Author contributions

JQ designed and implemented the experimental scheme. BH sorted the data and wrote the manuscript. QS, RW, XZ, and YY completed the data proofreading section. J-HW: writing – review and editing. All authors contributed to the article and approved the submitted version.

## Funding

This work was supported by the National Natural Science Foundation of China (Grant No. 32000125) and the doctoral scientific research foundation of Henan Normal University (Grant No. 5101049170188).

## Conflict of interest

The authors declare that the research was conducted in the absence of any commercial or financial relationships that could be construed as a potential conflict of interest.

## Publisher’s note

All claims expressed in this article are solely those of the authors and do not necessarily represent those of their affiliated organizations, or those of the publisher, the editors and the reviewers. Any product that may be evaluated in this article, or claim that may be made by its manufacturer, is not guaranteed or endorsed by the publisher.
